# An Important Suggestion in Milk Hygiene

**Published:** 1899-12

**Authors:** 


### COMMUNICATIONS.

---

### AN IMPORTANT SUGGESTION IN MILK HYGIENE.

THE idea of training cows to drop their manure in a shovel at more or less regular intervals may appear to be utopian, and yet the comparative ease with which "urination" and "defecation" in dogs and horses is subjected to control inclines me to the belief that something may be accomplished in this direction with even the less intelligent bovine species, whereby the manure nuisance in our dairy barns may be decidedly mitigated.

Those who are confronted with the numerous problems of dairy sanitation and milk hygiene will readily appreciate the drift of the scheme. Every one who has made even a superficial study of the habits of cows has observed how responsive is the act of defecation to the slightest disturbance, especially if they are touched in the neighborhood of the croup or tail, and that if a cow is lying down she will, on being made to stand, very frequently drop her manure. In testing cows for tuberculosis, both the insertion of the thermometer and the prick of the needle will induce an evacuation of the bowels in most cases, I believe, where the rectum is full.

I first tried to make cows dung by inserting a smooth, greased stick and injecting about three ounces of water by means of a small hard-rubber syringe, with the object of stimulating the anal sphincters and causing a desire to defecate. My observations, however, were too meagre to permit any reliable deductions from these methods.

I then instructed the night watchman in our dairy to make periodic trips, three each night for four consecutive nights, down one of the lines containing about twenty cows, and making those cows that were lying down rise. The following is a memorandum of the results:

| | | | |
|---|---|---|---|
| First night, | 47 recumbencies. | 15 evacuations. | 11 standing. |
| Second " | 45 " | 19 " | 11 " |
| Third " | 35 " | 15 " | 16 " |
| Fourth " | 42 " | 18 " | 15 " |
| Total, | 169 " | 67 " | 53 " |

There was a total of 169 recumbencies and 67 evacuations, or almost 40 per cent. of the cows, so to speak, lying down, when made to rise dropped their manure in a shovel without any attempt at training them to do so.

To what extent such a system can be made practicable and economical in the daytime as well as night—in fact, whenever cows are in the barn—would require a much more elaborate investigation than the above figures demonstrate; and yet any step that would contribute to the abatement of the manure nuisance, as it exists to-day in our dairies, would be welcomed by all who hold dear the welfare of our dairy interests. That it is unavoidable for a cow to indiscriminately drop her manure, and then lie in it until her rump, tail, and udder are plastered with it, seems to me absurd. If some of our sanitarians who are devoting their energies to the innumerable devices for freeing milk from the myriads of bacteria that it ordinarily contains would direct their thoughts to enforcing measures that would keep the milk sterile, as it practically is when it leaves the udder, it seems to me that milk hygiene would be planted on a more rational basis.

